# Tracking Cation Exchange in Individual Nanowires *via* Transistor Characterization

**DOI:** 10.1021/acsnano.4c05197

**Published:** 2024-06-25

**Authors:** Daniel Lengle, Maximilian Schwarz, Svenja Patjens, Michael E. Stuckelberger, Charlotte Ruhmlieb, Alf Mews, August Dorn

**Affiliations:** † Institute of Physical Chemistry, University of Hamburg, 20146 Hamburg, Germany; ‡ The Hamburg Center for Ultrafast Imaging, 22761 Hamburg, Germany; § Centre for X-ray and Nano Science CXNS, Deutsches Elektronen-Synchrotron DESY, 22607 Hamburg, Germany; ∥ Niedersachsen.next, 30159 Hannover, Germany

**Keywords:** Cation Exchange, CdSe, Ag_2_Se, Semiconductor Nanowires, Doping, Nanowire Field-Effect Transistors, Transistor Characterization

## Abstract

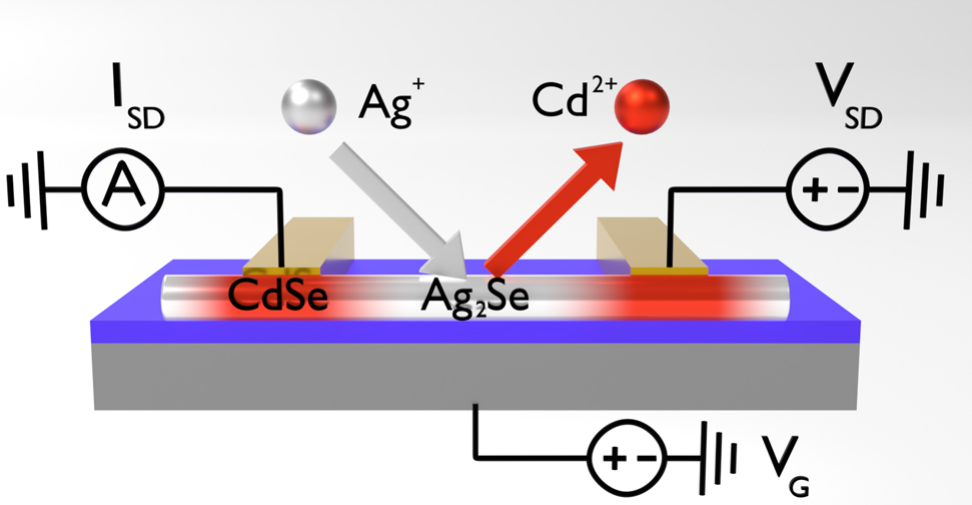

Cation exchange is a versatile method for modifying the material composition and properties of nanostructures. However, control of the degree of exchange and material properties is difficult at the single-particle level. Successive cation exchange from CdSe to Ag_2_Se has been utilized here on the same individual nanowires to monitor the change of electronic properties in field-effect transistor devices. The transistors were fabricated by direct synthesis of CdSe nanowires on prepatterned substrates followed by optical lithography. The devices were then subjected to cation exchange by submerging them in an exchange solution containing silver nitrate. By removal of the devices from solution and probing the electrical transport properties at different times, the change in electronic properties of individual nanowires could be monitored throughout the entire exchange reaction from CdSe to Ag_2_Se. Transistor characterization revealed that the electrical conductivity can be tuned by up to 8 orders of magnitude and the charge-carrier mobility by 7 orders of magnitude. While analysis of the material composition by energy dispersive X-ray spectroscopy confirmed successful cation exchange from CdSe to Ag_2_Se, X-ray fluorescence spectroscopy proved that cation exchange also took place below the contacts. The method presented here demonstrates an efficient way to tune the material composition and access the resulting properties nondestructively at the single-particle level. This approach can be readily applied to many other material systems and can be used to study the electrical properties of nanostructures as a function of material composition or to optimize nanostructure-based devices after fabrication.

Nanowires (NWs) offer high surface-to-volume ratios^1,2^ and can be made of different materials such as semiconductors,^1,2^ metals,^3^ superconductors,^4^ topological insulators,^5^ or thermoelectrics.^6^ Applications include solar cells,^7,8^ photodetectors,^9,10^ sensors,^11,12^ and field-effect transistors.^13,14^ Nanowire synthesis can be performed by physical methods such as molecular beam epitaxy^15,16^ or laser ablation^17^ as well as by chemical syntheses including chemical vapor deposition,^18^ vapor–liquid–solid,^19,20^ solution–liquid–solid,^21,22^ or solvothermal approaches.^23^ For most nanowire syntheses, catalyst particles are essential to facilitate NW growth and to control their diameter.^17−22^ The material properties and potential applications depend heavily on nanowire composition and crystal structure, which can be influenced during synthesis to achieve for example doping^24^ or alloying.^25^ In addition, heterostructures, *e.g*., core–shell^18^ or segmented^26^ structures, can be synthesized to tune electrical and optical properties.

However, not all geometries or compositions, especially doped or alloyed compositions, are available *via* direct synthesis. Using nanostructures with specific shapes and sizes as templates, cation exchange can be utilized to achieve the desired material composition.^27−29^ CdSe is an ideal template material, offering highly tunable opto-electronic properties based on various morphologies and structures accessible *via* a broad range of syntheses.^27−30^ During cation exchange, the nanomaterial morphology is preserved, as long as the reaction zone is smaller than the nanostructure, which is important when a specific geometry is required.^27^ Transforming CdSe nanowires into Ag_2_Se nanowires by cation exchange presents a convenient method not only to overcome the lack of wet chemical synthesis routes for Ag_2_Se NWs^31−33^ but also to tailor the material composition of the nanostructure.^34^

In this work, cation exchange from CdSe to Ag_2_Se is studied in nanowire field-effect transistors (NWFETs) by using individually contacted nanostructures. This model system demonstrates how the electrical conductivity can be tuned over 8 orders of magnitude by cation exchange in individual nanowires. Our approach allows us to monitor the electrical properties of single nanowires as a function of material composition without device-to-device variations due to geometry or device fabrication.

We performed nanowire synthesis directly on the substrates, which makes subsequent device fabrication easier, by avoiding additional nanowire deposition and difficulties with nanowire clustering. The procedure used in this work was adapted from Schwarz et al.^33^ (see Figure 1a). Degenerately doped silicon wafers with a 300 nm layer of insulating silicon oxide were used as substrates. Contact pads and catalyst patches were defined *via* optical lithography followed by physical vapor deposition (PVD). For the contact pads, we used a 10 nm titanium adhesion layer underneath a 50 nm gold layer. Catalyst patches were freshly deposited prior to CdSe nanowire synthesis and consisted of a 5 nm titanium/5 nm gold layer capped with a 20 nm bismuth layer. An optical microscope image of a bismuth patch prior to synthesis is shown in Figure 1b.

**Figure 1:**
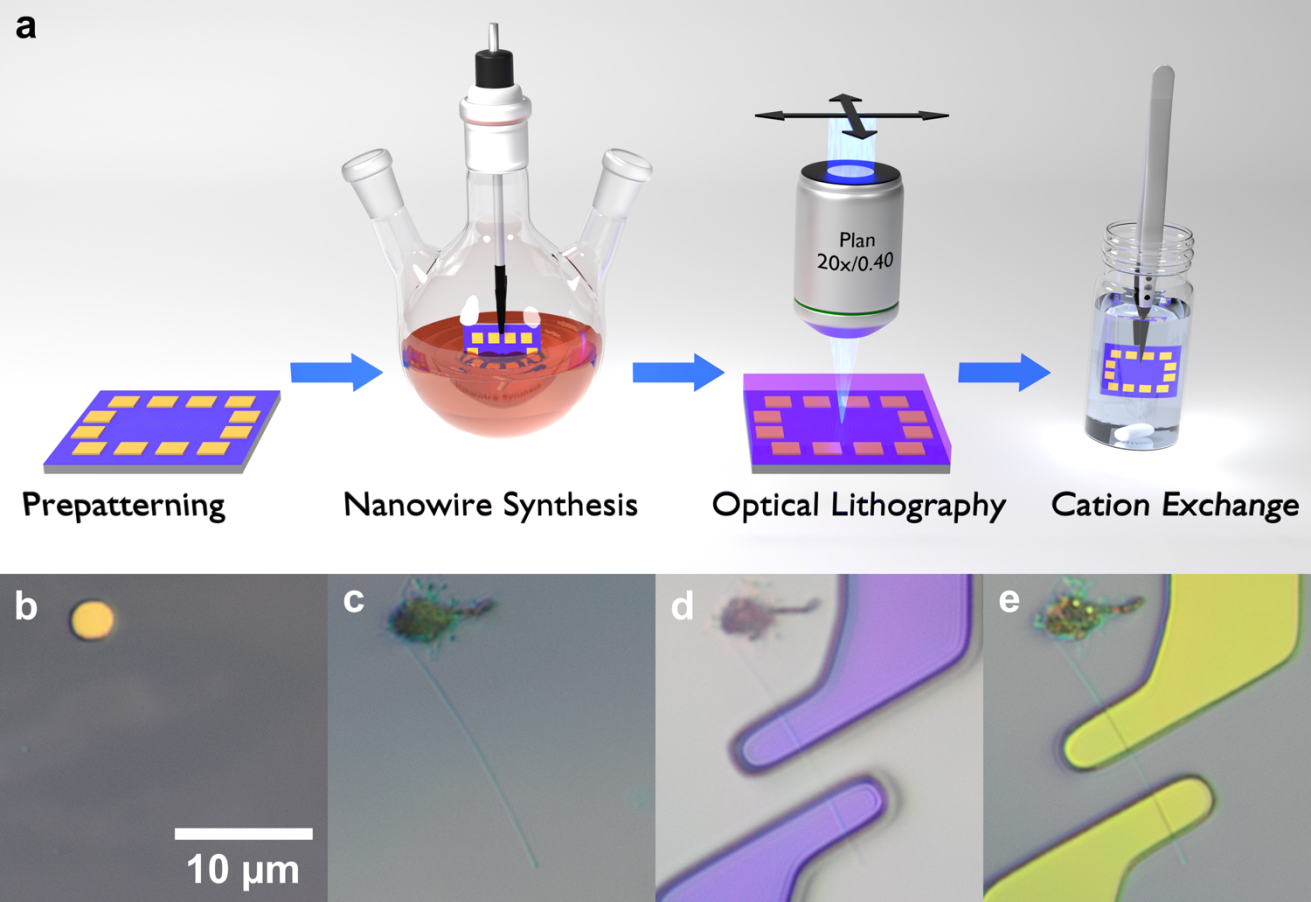
(a) Schematic overview of device fabrication. (b–e) Optical microscope images after (b) prepatterning, (c) synthesis, (d) lithography, and (e) lift-off.

CdSe nanowires were synthesized by a solution–liquid–solid^21^ method that was modified for direct synthesis on substrates^9^ (see Figure 1a). This synthetic procedure simplifies device fabrication by confining nanowire growth to a designated area defined by specific placement of the catalyst on the substrate. In addition, potential damage to the nanowires caused by ultrasonification commonly used to separate nanowires from one another can be avoided. The lithographically defined Bi patches break up into small Bi droplets upon heating and act as catalyst particles^9,33^ for nanowire growth. In addition, these catalyst particles fixed the nanowires in place on the substrate. This procedure also enables highly precise reaction timing, as substrates can be removed from the solution at any given time during the cation-exchange reaction and can be easily cleaned. Figure 1c shows an optical microscope image of a CdSe nanowire grown from a molten Bi patch. This synthesis typically yields nanowires that are 5 to 20 μm long with diameters around 30 nm. Using HRTEM in combination with X-ray diffraction data, a mixture of zincblende and wurtzite crystal structure was found, where the wurtzite phase dominates (see Supporting Information S1).

Nanowires can be contacted directly by optical lithography using a laser writer. A double-layer photoresist system was utilized to achieve an undercut resist profile after development, resulting in a clean lift-off process and thus a well-defined lead topography. To ensure an optimal contact between the nanowires and the metal contacts, the sample was exposed to a mild plasma cleaning step of 7.2 W for 30 s directly before metal deposition to remove potential photoresist residues and ligands from the wires under the contact area. Contacts to an NW after developing the photoresist and after deposition of 75 nm titanium are shown in Figure 1d,e, respectively. For electrical access to the back gate electrode formed by the Si substrate, the insulating SiO_2_ layer was removed at a corner of the substrate prior to metal deposition. To further improve the metal–semiconductor interface and remove remaining ligands and residue from lithography, devices were annealed in vacuum at 300 °C after lift-off.

Cation exchange from CdSe to Ag_2_Se was performed by submerging single nanowire transistor devices in a solution consisting of silver nitrate, methanol, and toluene^29,35^ for specified amounts of time, as shown in Figure 1a. This procedure allows for successive exchange processes on the same device and enables precise control over the reaction time and, therefore, the degree of cation exchange.

## Results and Discussion

### Electrical Properties as a Function of Cation Exchange

**Figure 2:**
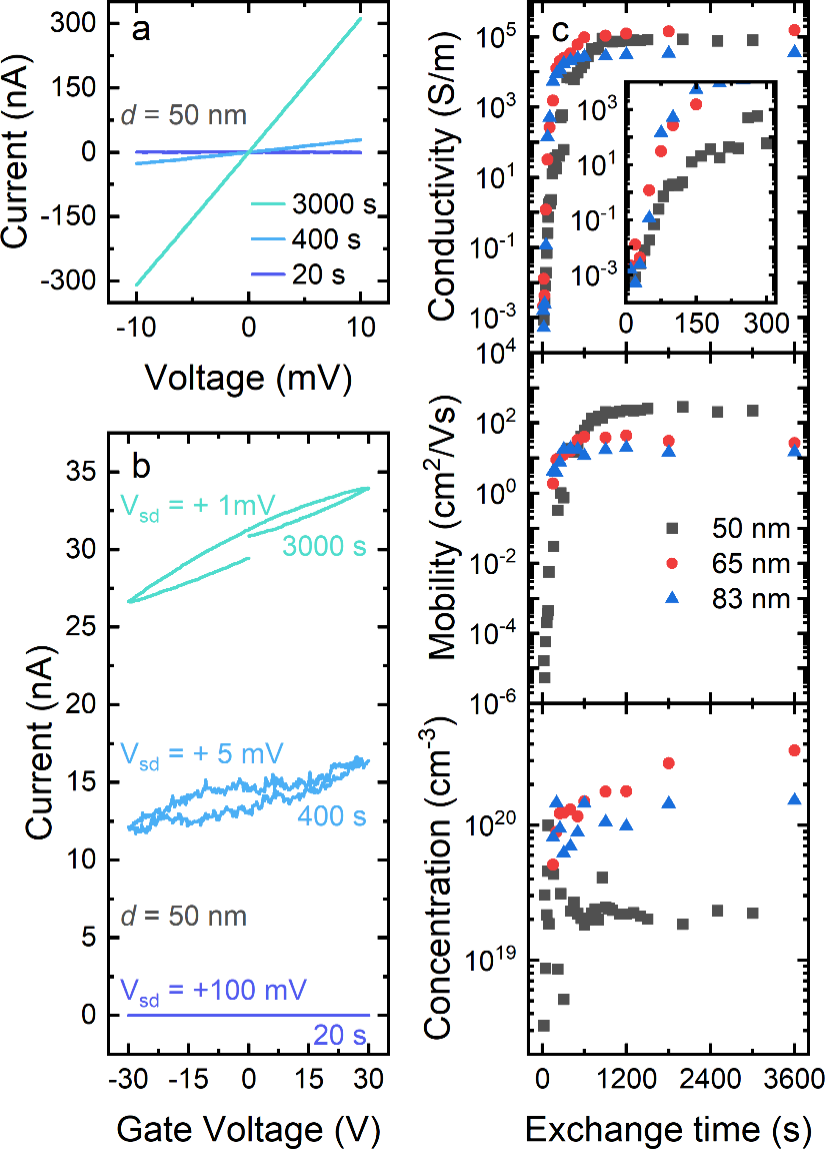
(a) *IV* curves and (b) transfer characteristics of a nanowire with a diameter of 50 nm after 20, 400, and 3000 s of cation exchange. All measurements were performed under ambient conditions in the dark. (c) Evolution of the specific conductivity σ, charge-carrier mobility μ_e_, and charge-carrier concentration *n*_e_ as a function of cation-exchange time from CdSe (0 s) to Ag_2_Se (3600 s) for three different nanowires with diameters of 50, 65, and 83 nm. The inset in the top panel shows a magnification of the first 300 s.

As-fabricated CdSe NWFETs were insulating to >50 GΩ, which was the resolution limit of our setup. The transport measurements were performed under ambient conditions in the dark to exclude the influence of photoinduced charge carriers. While there are reports that CdSe nanowires can degrade under illumination while exposed to oxygen^36^ and that CdS nanowires are sensitive to oxygen adsorption and humidity,^37^ we did not observe any influence or degradation during our measurement series. With ongoing cation exchange to Ag_2_Se, we observed an increase in the conductivity, as illustrated in Figure 2a. The specific conductivity σ of the nanowires was calculated according to:
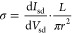
1where *L* is the length of the nanowire between the electrodes and *r* is the nanowire radius, both of which were obtained by atomic force microscopy (AFM, see Figure S2). The calculated nanowire conductivity was corrected for the resistance of the metal-contact leads. Potential contact resistance arising, *e.g.*, from Schottky barrier formation at the metal–nanowire interface was not taken into account.

The response to the gate voltage is shown in Figure 2b, indicating n-type majority charge carriers. The observed small hysteresis can be attributed to charge trapping in surface states.^38,39^ By applying a linear fit to the linear portion of the data the transconductance *g*_m_ = d*I*_sd_/d*V*_g_ was extracted.^38−40^ Using the transconductance *g*_m_, the charge-carrier mobility μ_e_ was estimated according to:^38−40^
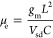
2where *C* is the capacitance between the nanowire and the gate electrode, which we obtained from finite element simulations utilizing COMSOL Multiphysics^41^ (see Figure S3). Using these results, the charge-carrier concentration *n*_e_ was calculated by:
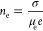
3where μ_e_ is the charge-carrier mobility and *e* is unit charge.

In Figure 2c, the evolution of the specific conductivity σ, charge-carrier mobility μ_e_ and charge-carrier concentration *n*_e_ is plotted as a function of cation-exchange time. Calculations were performed assuming homogeneous cation exchange within the entire NW, and the effective channel length, *i.e.*, length of the nanowire between the electrodes, and diameter were assumed to be constant as determined by atomic force microscopy.

A continuous increase in specific conductivity as a function of the cation-exchange time was observed. This suggests that cation exchange is homogeneous along the axis of the nanowires between the contacts.^29,35^ Dorn et al.^35^ observed topotaxial cation exchange from CdSe to Ag_2_Se in mats of nanowires by SEM and confirmed that the lattice spacing before and after cation exchange remains unchanged by HRTEM. This is consistent with our findings (see Figure S1) and with various reports which showed that cation exchange is expected to be homogeneous in larger nanostructures, if the lattice mismatch is small.^29,42,43^ On the other hand, if cation exchange proceeded from the electrode edges^44^ or formed segments,^45^ as reported for other systems, a sudden transition and consequently a jump in conductivity would be expected. Here, however, the conductivity increases continuously over 8 orders of magnitude from highly insulating (σ < 10^–3^ S/m) to conducting (σ ≈ 10^5^ S/m). This drastic increase in conductivity throughout the transition from insulating CdSe^9,46,47^ to conductive Ag_2_Se NWs^33,48^ renders this parameter particularly useful for monitoring cation exchange. The increase in conductivity is most pronounced during the first 600 s of the reaction and then slows down. After roughly 3600 s, specific conductivities of typically around 1·10^5^ S/m were obtained, which are comparable with literature values of 0.6·10^5^ S/m reported for Ag_2_Se NWs^48^ and (0.77–1.99)·10^5^ S/m for bulk Ag_2_Se.^49^

The charge-carrier mobility follows a trend similar to that of the specific conductivity. Starting at values below 10^–5^ cm^2^/V s, a continuous increase over 7 orders of magnitude up to values in the range of 10^1^–10^2^ cm^2^/V s was observed. This increase in mobility with progressing cation exchange can be explained by enhanced screening of charged impurities with increasing charge-carrier density.^50^ However, other effects, such as varying defect concentration or Fermi-level pinning at the nanowire surface, could also play a role. The values for charge-carrier mobility found here appear to be rather low when compared to other reports (10^0^–10^–1^ cm^2^/V s);^51^ however, in these reports, often thin films are investigated based on doped or modified CdSe nanowires. Additionally, it is often unclear if these measurements were performed under illumination where excited charge carriers can boost the electrical performance. Lower mobilities have been reported for pristine CdSe NWFETs ranging from 10^–1^ to 10^–4^ cm^2^/V s,^51,52^ especially for small individual nanowires low mobilities of 5·10^–4^ cm^2^/V s^53^ have been reported.

Sahu et al.^54^ report a charge-carrier type inversion at very low doping concentrations of 1–20 dopants per nanocrystal. So far, we have not been able to clearly identify p-type majority charge carriers at very low doping concentrations in our NW devices. This could be due to Schottky barriers and slower cation exchange below the contacts.

Literature values reported for the charge-carrier mobility of Ag_2_Se are about 1 to 2 orders of magnitude larger as compared to the values we obtained, and range from 0.85·10^3^ cm^2^/V s for Ag_2_Se NWs^48^ to 11.6·10^3^ cm^2^/V s for bulk Ag_2_Se.^49^ The lower charge-carrier mobilities in our nanowires potentially result from more pronounced surface scattering in the nanowire geometry and from crystal defects arising from twinning and zincblende/wurtzite mixtures in as-synthesized CdSe nanowires, which served as templates (see Figure S1).^39,55,56^ It is likely that additional defects were introduced by cation exchange, further lowering the mobility.^27,57,58^ Mild annealing can help to reduce the amount of defects,^27^*e.g.*, Schwarz et al.^33^ showed that after annealing the crystallinity of Ag_2_Se NWs increases, resulting in a more pronounced superionic phase transition.

The determined charge-carrier concentrations range from about 10^19^ to 10^20^ cm^–3^ and only span 1 order of magnitude. Starting already at a high level compared to literature values (0.4·10^19^ cm^–3^ for Ag_2_Se NWs^48^ and 0.11–0.15·10^19^ cm^–3^ for bulk Ag_2_Se^49^), the observed concentrations typically reach values of above 10^20^ cm^–3^. In particular for short durations of cation exchange, Schottky barrier formation at the contacts, the gate coupling effect, and weakly pronounced transfer characteristics can lead to significant uncertainties.^38,55,56,59−61^ This is further confirmed by the fact that, after 300 s, a more pronounced trend emerges, coinciding with an improved response to applied gate voltages. In other words, during early stages of cation exchange effects at the nanowire contacts likely result in an underestimated mobility and hence, according to eq. 3, in an overestimated charge-carrier concentration.^55,56^

Considering the mixed crystal structure of wurtzite and zincblende typically found in CdSe NWs, their potential impact on cation-exchange dynamics is of interest. There are reports that defects, such as stacking faults, interstitials, vacancies, and grain boundaries, can significantly influence the diffusion rate of cations in the host lattice.^28,62^ These diffusion pathways can be dominant and necessary to form heterostructures.^28,62,63^ For example Justo et al.^62^ identified stacking faults as potential nucleation sites for the formation of PbS/CdS heterostructures by cation exchange. Therefore, the stacking faults observed in our NWs could also have an influence on the exchange dynamics. However, silver ions are known to be very mobile in the CdSe host lattice and diffuse preferably over the interstitial sites of the Se^2–^ sublattice. Consequently, stacking faults are expected to be less important.^28,64^ Additional work is necessary to fully understand the influence of defects on the cation-exchange reaction and the resulting optoelectronic properties.

**Figure 3:**
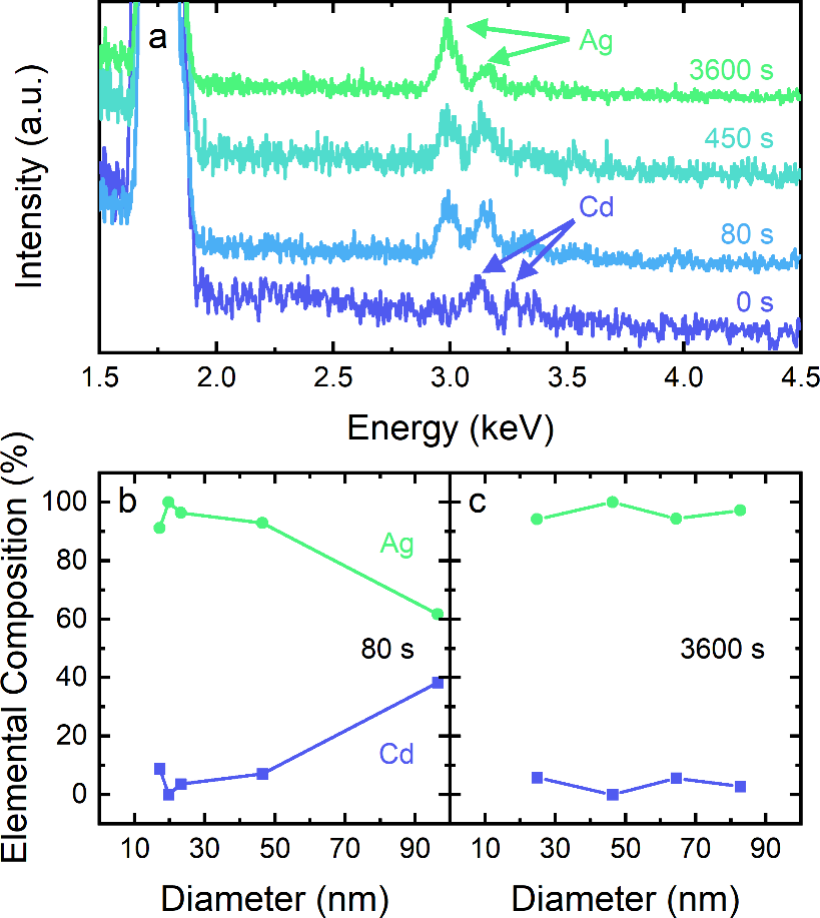
(a) EDX spectra of various CdSe/Ag_2_Se NWFETs exchanged for 0 s (*d* = 40 nm), 80 s (97 nm), 450 s (68 nm), and 3600 s (83 nm). (b,c) Measured cadmium and silver content in NWFETs exchanged for (b) 80 s and (c) 3600 s in relation to NW diameter.

Cation exchange is expected to take longer for NWs with larger diameters, resulting in a slower evolution of their electrical properties, such as the conductivity. The apparent absence of a distinct diameter dependence in Figure 2c could have multiple reasons but is most likely caused by variations in nominally similar, but different, fabrication cycles. This hypothesis is further confirmed in the section below, where a clear diameter dependence was observed for devices fabricated in the same run on the same substrate (Figure 3). While device-to-device variations caused by the fabrication process typically impede comparability, our approach circumvents this difficulty by allowing observation of cation exchange on the same nanowire for the entire series of reactions.

### Material Composition

Scanning electron microscopy (SEM) was used in combination with energy dispersive X-ray spectroscopy (EDX) to confirm the change in the material composition during cation exchange. EDX spectra of NWFETs subjected to cation exchange for different periods of time are shown in Figure 3a. Since single nanowires have small volumes compared to the macroscopic silicon substrates, a substantial background signal is prevalent. In addition, the Lα_1_ line of cadmium and the Lβ_1_ line of silver overlap,^65^ which makes an exact determination of the Cd and Ag content challenging. Due to the damage inflicted by the electron beam upon acquisition of EDX spectra, a fresh sample was used for every cation-exchange time. Even though samples were prepared nominally in the same way, variations can occur for various reasons, *e.g.*, incomplete removal of photoresist or chemical reactions on the nanowire surface. This can result in varying exchange rates.

From the recorded spectra in Figure 3a, the Cd and Ag contents were determined. The obtained elemental composition is plotted against the NW diameter for the sample that was exchanged for 80 s in Figure 3b and for 3600 s in Figure 3c. The first observation is that the exchange rate is dependent on NW diameter. Nanowires with large diameters (>40 nm) show a lower silver-to-cadmium ratio than NWs with small diameters (<40 nm) with close to 100% Ag content. This could be expected since the diffusion of Ag^+^ into and of Cd^2+^ out of the core of the NW is slower in thicker NWs with a lower surface-to-volume ratio. Figure 3c confirms complete cation exchange after 3600 s, as also indicated by transport measurements. Across all NW diameters a silver content above 95% was determined. Minor deviations can be explained by the difficulties of overlapping Cd and Ag signals and the poor signal-to-noise ratio based on the small cross-section of the nanowires. The second observation is that cation exchange appears to take place on a faster time scale than originally indicated by electrical transport measurements: while EDX spectra suggest significant silver content after 80 s, it is not until around 300 s into the cation-exchange reaction that a good electrical response emerges in transport measurements. In addition, it has to be taken into account that EDX was recorded only in the area between the contacts. As mentioned previously, cation exchange is expected to occur faster in the freely accessible segments between the metal contacts and is impeded for CdSe nanowire segments buried below the contact pads. Cation-exchange dynamics at and beneath the contacts therefore play a significant role for the evolution of the observed electrical properties during cation exchange.

**Figure 4:**
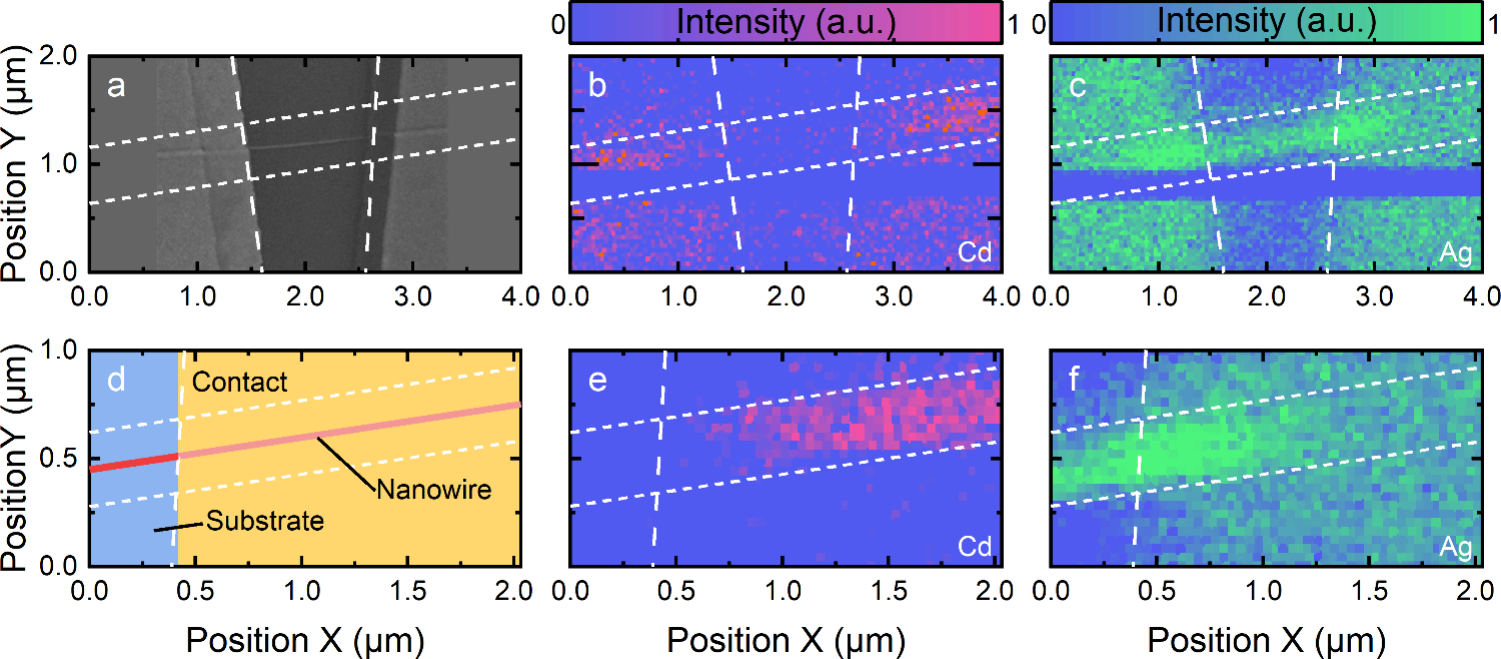
X-ray fluorescence of a partially exchanged NWFET. (a) SEM image of the investigated NWFET, scaled to fit the XRF maps. (b,c) XRF map of (b) Kα line intensity of cadmium and (c) silver of the device after background correction. (d) Schematic illustration of the right contact area. (e,f) XRF map of (e) Kα line intensity of cadmium and (f) silver of the right contact area with a higher resolution. To increase visibility, the intensities were normalized for each map individually. The section of low intensity in parts (b) and (c) results from a loss in excitation intensity during the measurement. The dashed line indicates the contact position, while the dotted line indicates the nanowire position; both were added as a guide to the eye. The measurements were performed with a beam energy of 28 keV (λ = 0.44 Å).

To verify cation exchange, especially below contacts, X-ray fluorescence (XRF) was recorded for a partially exchanged NWFET (25 mM AgNO_3_ (MeOH), for 30 s). An SEM image of the investigated device is shown in Figure 4a. XRF revealed that after cation exchange, Cd was absent and Ag was present in the entire nanowire segment between the contacts, as shown in Figure 4b,c, respectively. The section close to the contact and the “free” NW, schematically illustrated in figure 4d, is of particular interest to prove cation exchange below contacts. As shown in Figure 4e,f, an absence of cadmium (Figure 4e) and a presence of silver (Figure 4f) up to 300 nm below the contact were measured, suggesting that cation exchange also takes place beneath the metal film. The seemingly higher intensity of the Ag signal below the contacts can be attributed to the overlap of the Ag signal with a pronounced background caused by Compton scattering (high intensity in the whole contact region). The summed up XRF spectra of the scan (Figure 4b,c) are shown in the Supporting Information (Figure S4), to demonstrate intensity extraction.

These findings imply that Ag ions can diffuse along the axis of CdSe nanowires, which enables exchange underneath the metal contacts, allowing for the reaction to take place at the metal–semiconductor interface as well. In contrast, Dogan et al.^66^ report that copper ions are unable to diffuse in axial directions in CdSe nanowires into segments with nonaccessible surfaces.

### Analysis of Electrical Contacts to the Nanowires

The metal contacts to the nanowires play an essential role in electrical characterization. It is therefore important to keep in mind that the metal contacts inhibit direct cation exchange of the insulating segments of the CdSe nanowire directly below the contacts. In order to measure an increase in conductivity, it is therefore necessary for Ag^+^ ions to diffuse in and Cd^2+^ ions to diffuse out from under the contacts along the nanowire axis. The observed increase in conductivity during cation exchange as well as the XRF measurements shown above confirm the presence of this process. However, cation exchange in the nanowire segments beneath the metal-contact pads can still be expected to be slower than in the rest of the nanowire.

**Figure 5:**
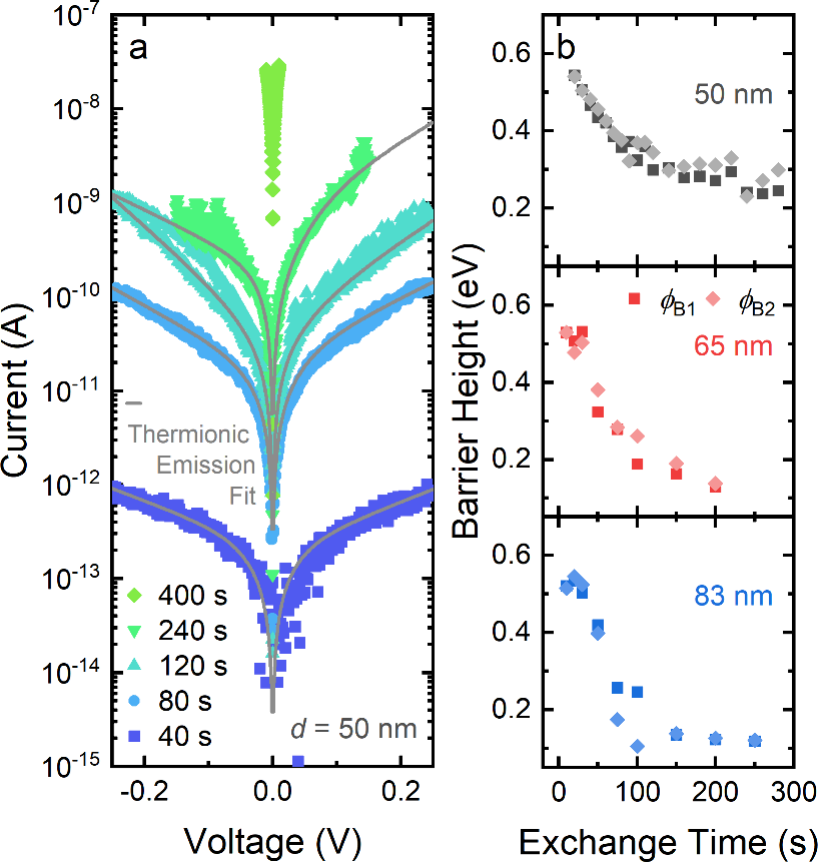
(a) Evolution of *IV* curves of an NWFET with a diameter of 50 nm during early stages of cation exchange including a fit based on the thermionic emission model, shown in gray. (b) Evolution of the calculated Schottky barrier heights as a function of cation exchange reaction time.

In Figure 5a, *IV* curves for a 50 nm NWFET are shown during the early exchange steps. The conductivity increases by 6 orders of magnitude during the first 400 s. Until about 300 s, the obtained *IV* curves show a nonlinear behavior indicating the presence of a potential barrier inhibiting charge-carrier injection from the metal into the semiconductor. The formation of a Schottky barrier at the metal–semiconductor interface concurs with reports regarding nanostructured devices,^59,60^ and can also help to explain the discrepancy in cation-exchange rate suggested by transport data and EDX measurements. After about 300 s, *IV* curves are linear, indicating Ohmic contacts. Since the nonlinear *IV* curves are not perfectly point symmetric, it is likely that the two contacts to the nanowire have slightly different barrier heights. In order to determine the barrier height of each contact individually, we employ a model for thermionic emission (see Figure 5a, more details concerning the employed model can be found in the Supporting Information S5).^67−69^

In Figure 5b, the calculated Schottky barrier heights of NWFETs for both contacts are shown as a function of the cation-exchange time. An initial barrier height of about 0.5 eV was found for all devices. Different work functions have been reported for CdSe: for bulk crystals values around 5 eV,^70,71^ for quantum dots above 4.4 eV,^72^ and for nanowires around 3.7 eV.^66^ Work functions also heavily depend on the surface chemistry, which should be kept in mind. While the reported value of 3.7 eV from Dogan et al.^66^ would explain a barrier formation with Ti (4.3 eV^67^), the barrier could also arise from segments of insulating CdSe NW buried below the contacts. With ongoing cation exchange, the barrier height decreases until about 300 s, after which linear *IV* curves were measured, indicating a transition from Schottky-like to Ohmic contacts. The continuous decrease in the barrier height indicates a gradual exchange of the contact region from CdSe to Ag_2_Se, consistent with the observed evolution of the conductivity discussed above.

To avoid Schottky barrier formation, we also fabricated devices with silver contacts. The barrier could then potentially be lowered by doping or partial cation exchange with silver at the metal–semiconductor interface. The fabricated devices, their transport characteristics, and elemental composition are shown in the Supporting Information S6. In short, the deposition of silver not only leads to complete cation exchange beneath the silver contacts but also within the entire nanowire between the contacts. This effect is likely facilitated by an increase in mobility of silver ions in the host lattice at elevated temperatures during the deposition process.

Combining the insights obtained from the transport and transfer characteristics, Schottky barrier height analysis, EDX and XRF measurements, three stages can be identified: In the first stage, until about 300 s, transport is dominated by nonlinear *IV* curves, indicating barrier formation between the nanowires and the metal-contact pads. With progressing cation exchange, the resistance and barrier height decrease continuously as Ag ions are able to diffuse below the contacts. In the second stage, starting after about 300 s up to 600 s, *IV* curves are linear, indicating Ohmic contacts, while the conductivity and mobility still increase significantly. In the third stage, after about 600 s until about 3600 s (complete exchange), only small changes in transport properties are observed. During this stage, most of the reaction has already taken place, and changes in transport properties are likely driven by purification from the removal of residual Cd ions.

## Conclusion and Outlook

In summary, we have demonstrated a versatile method for modifying the material composition of individual nanowires integrated into devices utilizing cation exchange. Our method allows for monitoring of the same nanowire in a field-effect transistor geometry over an entire cation-exchange reaction. The evolution of the specific conductivity and charge-carrier mobility were found to be continuous during the reaction from CdSe to Ag_2_Se, indicating homogeneous cation exchange within the nanowire. During the transition from practically insulating CdSe to conducting Ag_2_Se, nonlinear *IV* characteristics were observed in early stages of cation exchange, indicating Schottky barrier formation. This effect is likely influenced by the metal-contact pads impeding the cation exchange of CdSe nanowire segments directly below the contacts. Utilizing XRF, we were able to confirm that over time, cation exchange also takes place below the contact pads. After about 300 s, linear *IV* curves were observed, and cation exchange was typically complete after one hour.

**Figure 6:**
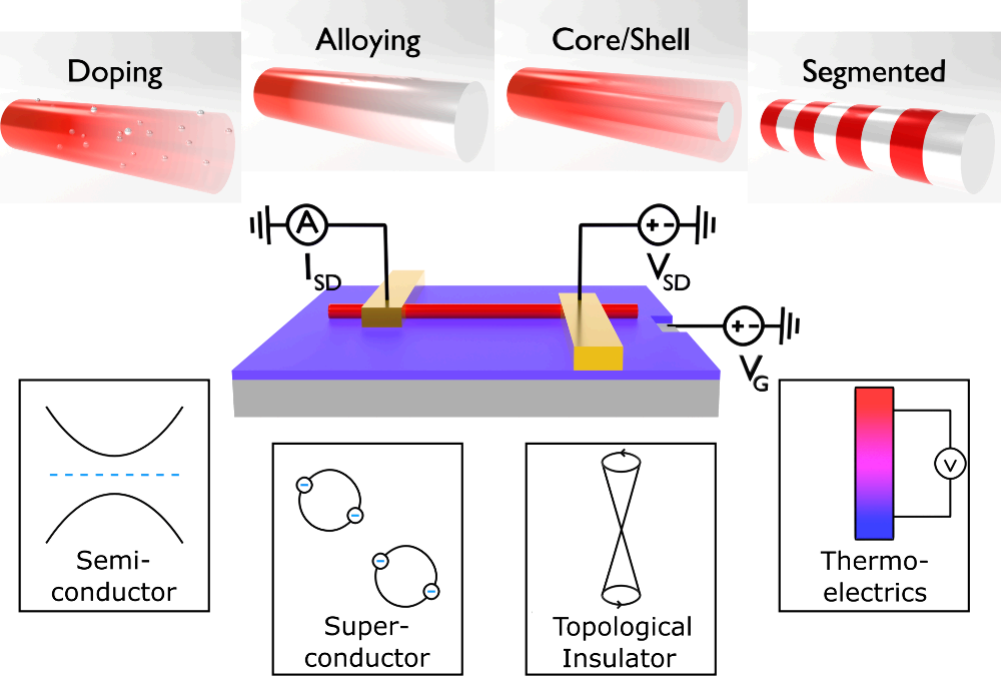
Schematic illustration of the use of single nanowire field-effect transistors in combination with cation exchange to investigate nanowires with various material compositions and properties.

Our method is especially beneficial for device optimization *via* cation exchange, since material properties can be directly controlled through the exchange time. This approach is not limited to the CdSe/Ag_2_Se system and can be applied to many other materials. The scientific potential of our method is illustrated in Figure 6. Cation exchange on single nanowire FETs can be used to efficiently study the properties of semiconductors, superconductors, topological insulators, or thermoelectrics as a function of the material composition on the same nanowire.

In addition, our method can be applied to material systems with different cation-exchange characteristics, including doping, alloying, core–shell structures, and the formation of domains or segments. Cation exchange on single nanowire field-effect transistors is a promising and very efficient strategy for studying the electronic properties of individual nanowires as a function of material composition, ultimately aiming at precise control of device properties at a single-particle level.

## Methods

### Chemicals

Acetone (C_3_H_6_O, VWR, >99.8%,), AZ ECI 3012 (Photoresist, MicroChemicals), AZ MIF 726 (Developer, Microchemicals), Bismuth (Bi, Chempur, 99.999%), Cadmium oxide (CdO, Chempur, 99.999%), Gold (Au, PIM, 99.99%), Hexane (C_6_H_14_, VWR, 98%), Isopropanol (C_3_H_8_O, VWR, >99.7%), LOR 5A (Lift-off resist, MicroChem), Methanol (CH_4_O, Grüssing, 99.5%), MICROPOSIT MF-319 (Developer, DuPont), MICROPOSIT Remover 1165 (Remover, DuPont), MICROPOSIT S1805 (Photoresist, DuPont), Octanoic acid (C_8_H_16_O_2_, Sigma Aldrich, >99%), Selenium (Se, Acros Organics, 99.5%), Silver nitrate (AgNO_3_, Sigma Aldrich, 99.9999%), Titanium (Ti, Alfa Aesar, 99.995%), Toluene (C_7_H_8_, Fisher Chemicals, 99.8%), Trioctylphosphine (C_24_H_51_P, ABCR, 97%), Trioctylphosphine oxide (C_24_H_51_OP, Sigma Aldrich, 99%). All chemicals were used without further purification.

### Prepatterning

The general device prepatterning and fabrication are adapted from Schwarz et al.^33^ First, silicon wafers (500 μm, 300 nm SiO_2_, SI-Tech. Inc., Prime [ρ < 0.005], N/As) were thoroughly cleaned using acetone, isopropanol, and deionized water in combination with ultrasonication, spin-coated with photoresist (AZ ECI 3012), and exposed with a mask aligner (MJB3, Karl Süss). After development (AZ MIF 726), 10 nm of Ti followed by 50 nm of Au were deposited *via* physical vapor deposition (PVD). Lift-off was carried out in acetone. This lithographic procedure was repeated for the Bi patches by depositing 5 nm Ti and 5 nm Au. The 20 nm bismuth layer was deposited immediately prior synthesis.

### Synthesis

The solution–liquid–solid nanowire synthesis directly on a substrate is derived from Littig et al.^9^ After bismuth deposition, lift off was carried out in acetone. The prepatterned substrate is then placed in a four-neck flask loaded with 20 g of trioctylphosphine oxide. At 120 °C, residual water and oxygen are removed under vacuum for at least 1 h. After heating up to 255 °C, the sample is submerged in the reaction solution and is given 60 s for the bismuth to melt. The cadmium precursor (Cd(OCA)_2_ dispersed in TOP, 0.15 mmol) was added first, followed by a slow injection (over 60 s) of the selenium precursor (Se-TOP, 0.15 mmol). After 10 min of reaction, the substrate is removed from the reaction solution. The substrate is then rinsed with toluene and hexane, washed with isopropanol, and then carefully dried under nitrogen flow. Cd and Se compounds are highly toxic and carcinogenic and must be handled with special care.

### Lithography

First the lift-off resist (LOR 5A) was spin-coated onto the substrates, followed by the photoresist (S1805). Contacts to the nanowire were defined by a laser writer (ML3, Durham Magneto Optics) with a dose of ∼130 mJ/cm^2^. After development (MF-319), the substrates were treated with mild air plasma (PDC-002, Harrick Plasma) of 7.2 W for 30 s and 75 nm Ti was deposited *via* PVD. Afterward, the substrates were submerged in the remover (Remover 1165) for typically 3 h and then cleaned in acetone and isopropanol followed by drying with nitrogen. Finally, the devices were annealed in vacuum at 150 and 300 °C for 30 min each and then exposed to UV irradiation for 20 min.

### Cation Exchange

First, a stock exchange solution was prepared by dissolving 8.5 mg of AgNO_3_ (0.05 mmol) in 10 mL of methanol. For cation exchange, 80 μL of this solution was added to a solution of 2 mL toluene and 8 mL methanol.^29,35^ The cation-exchange reaction was started by submerging the substrate into the solution and stopped after the desired time by removing it and rinsing with methanol and isopropanol. The reaction vessel was wrapped in aluminum foil and covered to reduce light induced reactions.

### Characterization

**Atomic force microscopy** was carried out with a JPK Nanowizard II instrument without any additional sample preparation always prior to cation exchange. Data processing and analysis was done with Gwyddion and MATLAB. **Scanning electron microscopy** and **Energy dispersive X-ray spectroscopy** were performed using a Zeiss LEO Gemini 1550 with beam energies ranging from 5 to 22 keV. **Transport measurements** were performed in a self-built probe station. The sample was placed in a grounded metal container and contacted with tungsten probe needles *via* micromanipulators (FormFactor, DPP105). The voltages to source, drain, and gate electrodes were applied with voltage source units (Yokogawa, GS200). To protect the sample and system in case of a failure of the dielectric layer, a 10 kΩ series resistance was connected to the gate electrode. The currents were measured with a multimeter (Keysight 34465A) in combination with a current amplifier (Femto, DLPCA-200). All measurements were performed under ambient conditions in the dark. The measurements were automated using a self-written LabView program. **X-ray fluorescence measurements** were performed at the microprobe endstation of P06 at PETRA III (DESY, Germany).^73^ The coherent fraction of the photon beam with an energy of 28.0 keV (above the AgK absorption edge) was focused to 100 · 100 nm^2^ (horizontal · vertical, fwhm) using Be compound refractive lenses (CRL) and a corrective phase plate.^74^ The fluorescence photons were collected using two silicon drift detectors (SII Vortex EM, Hitachi High-Tech Science Corporation, Tokyo, Japan) read-out by a digital pulse processor (Xspress3, Quantum Detectors, Harwell Oxford, UK) positioned inboard of the sample at angles of α_upstream_ = 56.4° and α_downstream_ = 113.2° with respect to the incoming beam.

## Supporting Information Available

Details concerning the crystal structure of the CdSe and Ag_2_Se nanowires including HRTEM images (S1); an exemplary AFM scan for geometry retrieval (S2); the COMSOL Multiphysics model for capacitance simulation and comparison with analytical methods (S3); illustration of XRF spectra used for XRF maps (S4); the thermionic transport model used to calculate Schottky barrier heights (S5); measurements on devices with silver contacts, including transport characteristics and determination of elemental composition (S6) (PDF)
