# Supplementary material for: Tracking Cation Exchange in Individual Nanowires via Transistor Characterization
Source: ACS Nano. 2024 Jun 25;18(27):18036–45. doi: 10.1021/acsnano.4c05197 (PMC11238621; doi:10.1021/acsnano.4c05197)
Supplement: Supplementary file 1 — nn4c05197_si_001.pdf [file nn4c05197_si_001.pdf]

Supporting Information for:

Tracking Cation Exchange in Individual Nanowires *via* Transistor Characterization

Daniel Lengle[a,b†], *Maximilian Schwarz*[a†], *Svenja Patjens*[a,c], *Michael E. Stuckelberger*[c], *Charlotte Ruhmlieb*[a], *Alf Mews*[a,b*] *and August Dorn*[d*].

[a] Institute of Physical Chemistry, University of Hamburg, 20146 Hamburg, Germany.

[b] The Hamburg Center for Ultrafast Imaging, 22761 Hamburg, Germany.

[c] Centre for X-Ray and Nano Science CXNS, Deutsches Elektronen-Synchrotron DESY, 22607 Hamburg, Germany.

[d]Niedersachsen.next, 30159 Hannover, Germany.

[*] Email: alf.mews@uni-hamburg.de, august.dorn@gmx.de

† D. L. and M.S. contributed equally to this paper

S1 Crystal structure of nanowires

To determine the morphology and crystal structure of the as grown CdSe nanowires a high-resolution transmission electron microscopy (HRTEM) investigation was performed. Images are shown in figure S1 (a) and (c) along with close-ups of specific areas (white box) in (b) and (d), respectively. The images reveal a high crystallinity of the nanowires as well as defects commonly observed in CdSe nanowires, such as stacking faults and twinning.[1–3] The spacing of the lattice planes in figure S1 (b) is about of 3.54 Å, which corresponds to the (002) direction (3.51 Å,[4] ICSD: 415786) of wurtzite CdSe and matches the typical growth direction of CdSe nanowires.[1–3] Perpendicular to the (002) plane the (100) plane can be observed in figure S1 (d) with a lattice spacing of 3.75 Å nm (3.72 Å,[4] ICSD: 415786). After complete cation exchange to Ag_2Se the high crystallinity is preserved, as can be seen in figure S1 (e) and (g), although some new defects have formed. Analysis of the lattice planes in figure S1 (f) and (h) indicate an orthorhombic crystal structure. While the determined spacing of 6.95 Å in figure S1 (f) can be assigned to the (010) plane (7.06 Å,[5] ICSD: 261822) the spacing of 2.57 Å can be attributed to the (121) plane of an orthorhombic lattice (2.59 Å,[5] ICSD: 261822). Nevertheless, the assigned (010) plane (6.95 Å) could also be interpreted as a (001) plane (7.02 Å,[4] ICSD: 415786) of the hexagonal CdSe host lattice and the (121) plane (2.57 Å) as a (102) plane (2.55 Å,[4] ICSD: 415786).

These findings are in accordance with Li *et al.*,[6] where they found that the hexagonal crystal structure of CdSe is maintained after cation exchange to Cu_2Se, even though a hexagonal structure of Cu_2Se is unusual, and was described as highly unstable. In addition, in a previous report by Dorn *et al.*[7] the nanowire geometry was also determined to be maintained after cation exchange from CdSe to Ag_2Se. They performed a HRTEM investigation after complete cation exchange to Ag_2Se to verify the topotaxial nature of the reaction. Indeed, the lattice spacing observed for Ag_2Se

nanowires fits the previously determined lattice spacing of CdSe. We therefore conclude that the crystal structure after cation exchange likely consists of both a hexagonal CdSe crystal structure and an orthorhombic Ag_2Se structure.

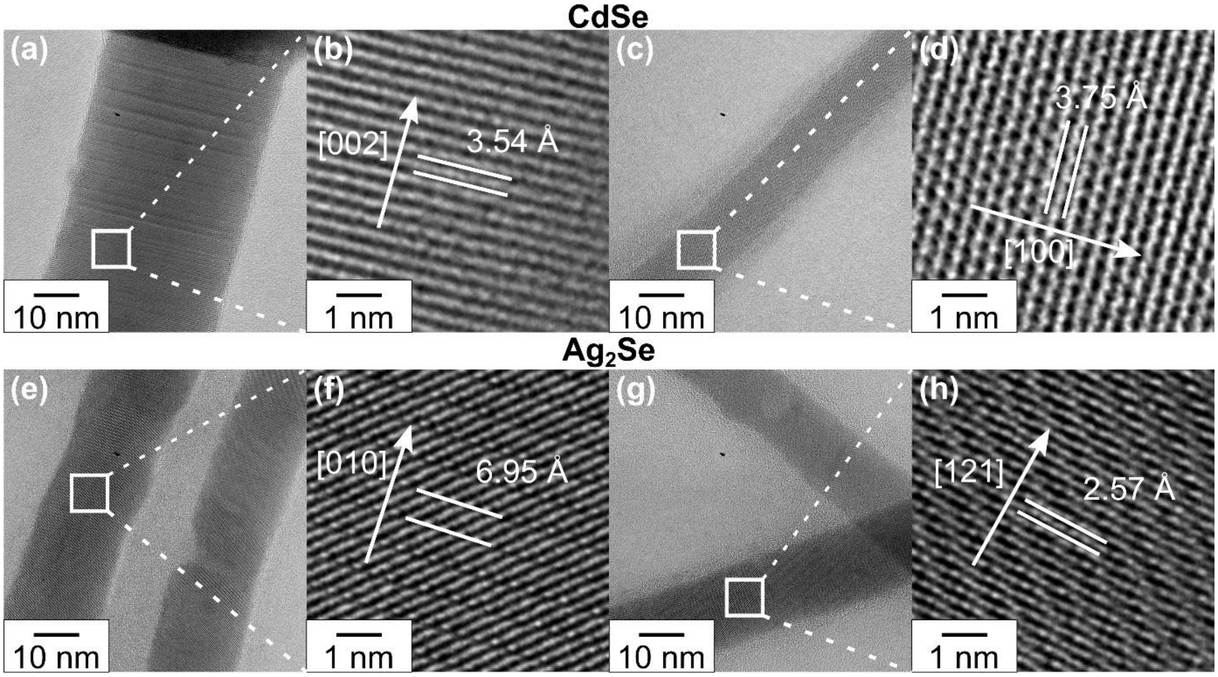

Figure S1. High resolution TEM images of (a–d) CdSe and (e–h) Ag_2Se nanowires. (b, d, f, h) Close-up of the marked areas of the images shown to visualize the lattice planes. The determined distance between two lattice planes as well as the direction are added for clarity.

Previously, we have further confirmed removal of cadmium throughout cation exchange by X-ray diffraction (XRD).[8] For this purpose, nanowires were grown as a dense film on a silicon wafer similar to the standard synthesis. To identify reflexes originating from the substrate, it was measured prior to synthesis as well. For the Ag_2Se NWs, cation exchange was carried out on a similar sample of CdSe NWs with an increased amount of $AgNO_3$ to compensate for the increased number of NWs and therefore to ensure comparability. The data can be found in the supporting information of the work of Schwarz *et al.*[8], here we recap the main findings.

Most reflexes present in the CdSe sample originate from the crystalline silicon wafer. The absence of most reflexes corresponding to CdSe can be explained by the dominant orientation effect as all nanowires lay flat on the substrate, resulting in an underrepresentation of reflexes in growth direction.[9] The few reflexes present at 23.8 ° and 42.0 ° were attributed to the wurtzite structure of CdSe. Since the reflex at 42.0 ° can be assigned to the zincblende structure of CdSe as well, together with the absence of other reflexes corresponding to zincblende and the insights from HRTEM, it is concluded that NWs exhibit predominantly wurtzite crystal structure with an admixture of zincblende. Due to an absence of reflexes it was not possible to determine the exact wurtzite-zincblende ratio as shown by Harder *et* al.[3] Noteworthy is that the reflexes at 27.0 ° (overlapping with wurtzite) and 39.5 ° were assigned to bismuth and most likely result from the recrystallization of the catalyst after melting under synthesis conditions.

After cation exchange to Ag_2Se, all reflexes previously assigned to the wafer and bismuth are still present. Furthermore, all reflexes corresponding to wurtzite CdSe are absent, indicating complete cation exchange. Similarly, only few reflexes were observed due to the orientation of the NWs on the substrate. The absence of any peaks indicating the presence of Ag_2Se could be a sign of disorder of the cations[10] or stacking faults[11] introduced after cation exchange as observed for various exchange reactions. For Ag_2Se, the orthorhombic phase has been observed in HRTEM for nanowires with diameters of 40 nm and larger and is believed to be the most stable phase.[12]

S2 Determination of nanowire diameter and channel length

The nanowire diameter and channel length of the fabricated CdSe nanowire field-effect transistors (NWFETs) were determined by atomic force microscopy (AFM). All AFM scans were performed prior to cation exchange to avoid interactions with potential residues from cation exchange.

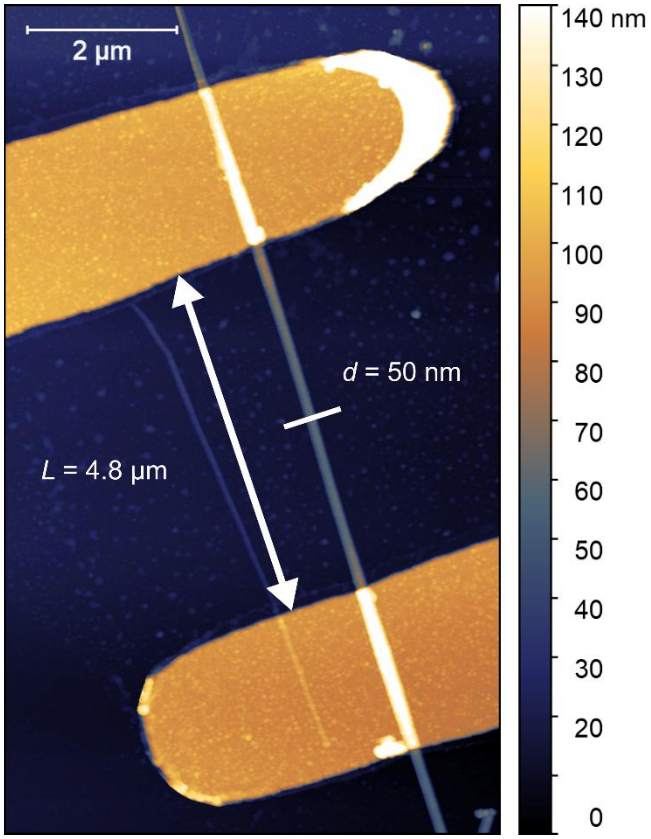

Figure S2. AFM scan of a CdSe-NWFET prior to cation exchange. The nanowire diameter is extracted *via* averaging over several line profiles across the nanowire as marked exemplary in white with the corresponding diameter determined. The channel length is obtained by measuring the distance between the electrodes along the nanowire, as indicated with the double arrow (with an offset to keep the nanowire visible) and the determined length. Note that the thinner second wire is not contacted on both sides and therefore does not contribute to charge transport.

The resolution of an AFM is best in the z-axis (height). Nanowire diameters were determined by averaging over 15 cross-sections of the nanowire in different places, as illustrated in figure S2. For each of these line profiles the background height was fitted and subtracted, and the maximum height was determined. The average of the maxima was calculated and used as nanowire diameter. For the channel length, the shortest distance between the electrodes along the nanowire was measured (double arrow in figure S2). For a reference sample the nanowire diameter and channel length were additionally determined by scanning electron microcopy (SEM). No significant deviations between SEM and AFM were observed, confirming AFM as a suitable and non-destructive characterization method.

S3 Simulation of the capacitance of nanowire field effect transistors

To determine the charge-carrier mobility μ_{e}, the capacitive coupling of the nanowire to the backgate C must be obtained first. In literature, the capacitance is often calculated by using an analytic model for a metallic wire over an infinite metal plate:[13,14]

$$C = \frac{2\pi\epsilon_{\mathrm{r}}\epsilon_0 L}{\ln\left(\frac{2h}{r_{\mathrm{NW}}}\right)} \tag{S1}$$

where ϵ_{r} is the dielectric constant of the surrounding medium, ϵ_0 is vacuum permittivity, L the channel length, h the distance between nanowire center and gate electrode and r_{NW} is the nanowire radius. This approach assumes that the nanowire is fully surrounded by the dielectric medium (in our case SiO_2) instead of a NW lying on top of the dielectric medium (SiO_2) with a second dielectric present (Air).[13,14] The NW is assumed to be infinite to neglect fringe fields at the electrodes and both gate and nanowire are assumed to be metallic to limit charge accumulation to

the surface.[13,14] These simplifications lead to significant discrepancies with more detailed models.[13,14]

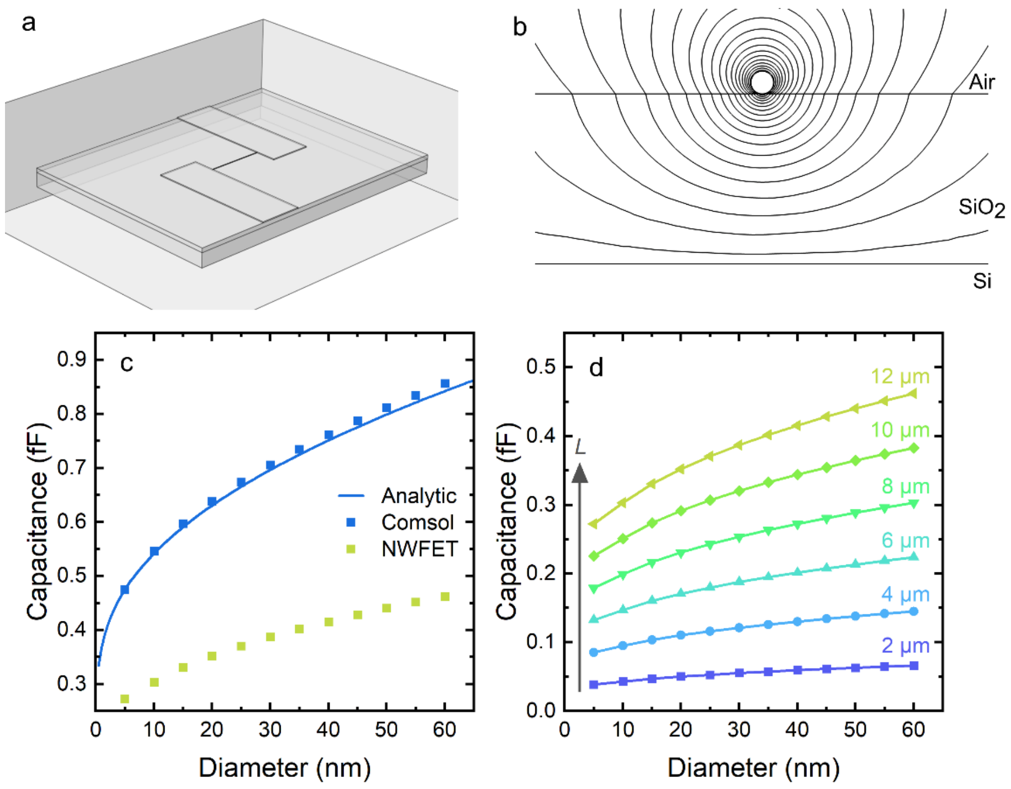

Figure S3. (a) COMSOL Multiphysics simulation model used for capacitance estimations. (b) Cross section through the 3D model to illustrate the electric-field lines. (c) Comparison between calculated capacitance for various nanowire diameters and a fixed channel length of 12 µm, using an analytical model according to eq. S1 (solid blue line) and COMSOL Multiphysics simulations (blue and yellow dots). The blue dots represent simulations following the assumptions made in the analytic model (in particular, the nanowire is completely surrounded by the dielectric). The yellow dots were obtained by simulating the NWFETs as depicted in (a). (d) Effect of the channel length on the capacitance as a function of the nanowire diameter, for channel lengths ranging from 2 µm (violet) to 12 µm (yellow).

To obtain values that more precisely reflect the geometry of our samples, COMSOL Multiphysics was used to model and simulate the capacitance of the NWFETs we studied. In order to demonstrate the feasibility of this approach, a model based on the metallic wire over an infinite metal plate model was simulated. The analytic solution (blue line) is compared with the results from the simulation (blue dots) in figure S3 (c) and shows good agreement. Next, the model was refined to match our experimental configuration with the NW lying on top of SiO_2 and surrounded by air as illustrated in figure S3 (a) and (b). The resulting capacitance is significantly smaller than the values obtained from the analytic model, since fringe fields at the contacts and the SiO_2/air interface are taken into account. In addition, in figures S3 (d) the influence of the channel length on the capacitance is demonstrated. With decreasing channel length, the influence of the fringe fields becomes more prominent, resulting in a lower capacitance. Since the NWFET geometry has a significant influence on the capacitance, as shown in figure S3, it was simulated for the dimensions of each device we studied. While most of the effects are considered in this approach, it has to be noted that the nanowire is assumed to be metallic. Since the NWs have a sufficiently high charge-carrier concentration (> 10^{18} cm^{-3}), this assumption appears reasonable.[13]

S4 X-ray fluorescence spectra

In order to verify cation exchange, especially below the contacts, X-ray fluorescence (XRF) was recorded. The XRF maps shown in figure 4 were obtained by extracting the corresponding Kα line intensity of cadmium (23.2 keV)[15] and silver (22.2 keV)[15] of the XRF spectra at each pixel. To illustrate such a XRF spectra, the spectra summed up across the entire scan of figure 4 (b) and (c) is shown in figure S4.

Besides the signals of interest for cadmium and silver between 22 and 24 keV, signals in the lower energy regime can be found and attributed to various other elements present on the substrate. Prominent peaks around 25.8 and 28.0 keV can be assigned to Compton and Rayleigh scattering, respectively. For better illustration of the Cd and Ag Kα peaks in the inset, the baseline was subtracted from the spectra and smoothed.

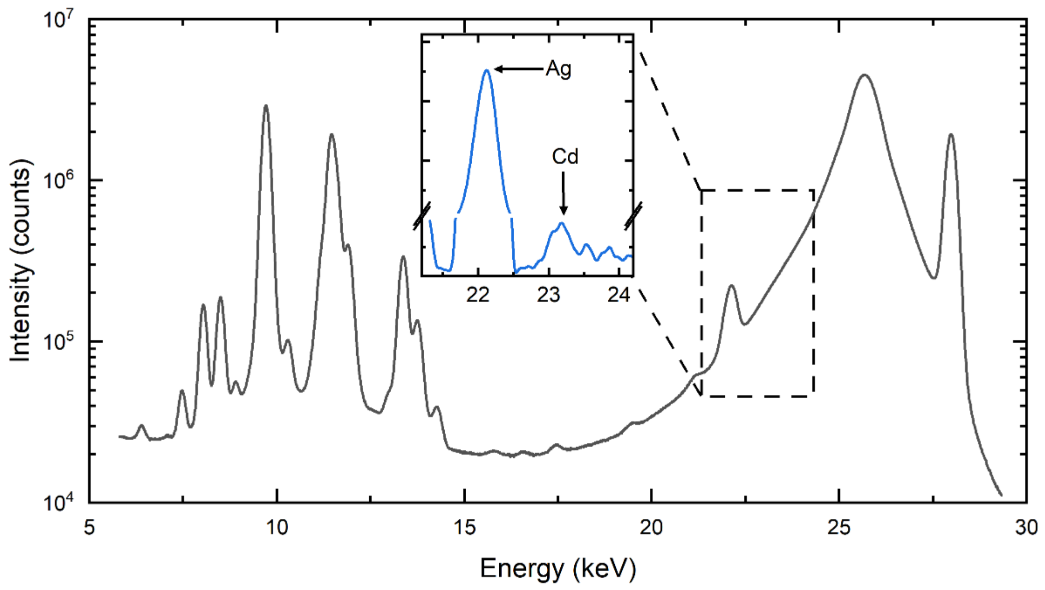

Figure S4. Raw XRF spectrum summed up of the scan shown in figure 4b and c. The inset shows the curve after baseline correction and smoothing. The points of intensity retrieval for the XRF maps for the cadmium and silver Kα peak are indicated by arrows.

S5 Thermionic transport model

Non-linear *IV* curves commonly originate from Schottky barriers at the metal–semiconductor interface.[16] Electrons can pass this barrier from the semiconductor into the metal dominantly either by thermionic emission or by tunneling.[16] For most semiconductors at room temperature the thermionic emission model is presumed to be the governing transport process.[16,17] The current density J then can be described by:[16,17]

$$
\begin{aligned}
J &= \frac{4\pi q m^* k^2}{h^3} T^2 \exp\left(-\frac{q\phi_\mathrm{B}}{kT}\right)\left[\exp\left(\frac{qV}{nkT}\right) - 1\right] \\
&= A^* T^2 \exp\left(-\frac{q\phi_\mathrm{B}}{kT}\right)\left[\exp\left(\frac{qV}{nkT}\right) - 1\right] \\
&= J_\mathrm{S}\left[\exp\left(\frac{qV}{nkT}\right) - 1\right]
\end{aligned} \tag{1}
$$

where q is unit electric charge, m^* the effective mass of the charge carriers, k the Boltzmann constant, h is Planck's constant, T the absolute temperature, ϕ_B the Schottky-barrier height, V the applied potential and n the ideality factor. The constants in the first part are usually summarized in the Richardson constant A^* and by introduction of the saturation current density J_S, eq. 1 can be further simplified.[16,17]

A NWFET can be described by two metal–semiconductor interfaces (Schottky diodes) connected back-to-back, therefore the net current of the device is given by the sum of both diodes. Eq. 1 can then be applied to this metal–semiconductor–metal case, giving:[17]

$$
J = \frac{2J_{\mathrm{S}1}J_{\mathrm{S}2}\sinh\left(\frac{qV}{2nkT}\right)}{J_{\mathrm{S}1}\exp\left(-\frac{qV}{2nkT}\right) + J_{\mathrm{S}2}\exp\left(\frac{qV}{2nkT}\right)}. \tag{2}
$$

For the sake of simplicity and comprehensiveness we would like to refer to Nouchi[17] who has presented a very detailed derivation of this equation. Both, image-force lowering caused by the applied potential as well as the simultaneous occurrence of forward and reverse current condition need to be taken into account when calculating the Schottky barrier, resulting in the following equation for the barrier height:[17,18]

$$\phi_{\mathrm{B1}} = \phi_{\mathrm{B01}} + V\left(\frac{1}{n_1} - 1\right)_{\text{(reverse)}} \tag{3}$$

$$\phi_{\mathrm{B2}} = \phi_{\mathrm{B02}} + V\left(1 - \frac{1}{n_2}\right)_{\text{(forward)}}$$

thereby ϕ_{B1} is the effective Schottky-barrier height and ϕ_{B01} is the barrier height at zero bias for contact 1 and equally for ϕ_{B2}, ϕ_{B02} for contact 2.[17,18]

The effective Schottky-barrier heights ϕ_{B1}, ϕ_{B2} were retrieved by fitting eq. 2 to the measured *IV* curves together with the adjustments of eq. 3. To convert the current density J to the measured current I the relation $I = J \cdot A$ was used with the assumption that the contact area equals the cross-sectional area of the nanowire A.[19] Based on the observation that the Ag_2Se fraction is mainly responsible for charge-carrier transport the effective electrons mass m^* of Ag_2Se ($m^* = 0.32\ m_0$)[20] was used to calculate the Richardson constant A^*. It has to be noted that this approach rests on many assumptions, including that the voltage drops only at the metal–semiconductor interface, which neglects a resistive contribution from the nanowire. Furthermore, the ideality factors n_1, n_2 were used to describe the asymmetry of contacts (in eq. 3) while the ideality factor n (in eq. 2) was set to one which excludes the influence of tunneling current. Wen *et al.*[21] proposed an even more detailed model to consider the influence of thermionic as well as tunneling current, which would

go beyond the scope of this analysis. Nevertheless, the applied model is capable of giving an insight into the change of barrier heights with progressing cation exchange.

S6 Introducing silver into contacts

As cation exchange is impeded below the contacts, a potential approach to improve the device response to cation exchange is to partially exchange the metal–semiconductor interface with silver. The first approach would be to perform a short cation-exchange reaction after lithography prior to metal deposition. However, this is not suitable since the solvents (methanol and toluene) cause a swelling and partial removal of the photoresist. More importantly, the exchange reaction would not be limited to the contact area, given the high mobility of silver ions within the CdSe matrix, resulting in a device with unknown material composition at the beginning of the experiment.

A more suitable approach would be to use silver as contact material. By mild annealing silver could then diffuse into the metal–semiconductor interface leading to a soft doping or exchange of the interface, and ultimately to a lowering of the barrier. We fabricated a device with narrow contacts containing 10 nm Ti as an adhesion layer and a broader 50 nm Ag contact layer (see inset in fig. S6 a), resulting in pattern allowing silver to be in direct contact with the NW, as shown in figure S6 a.

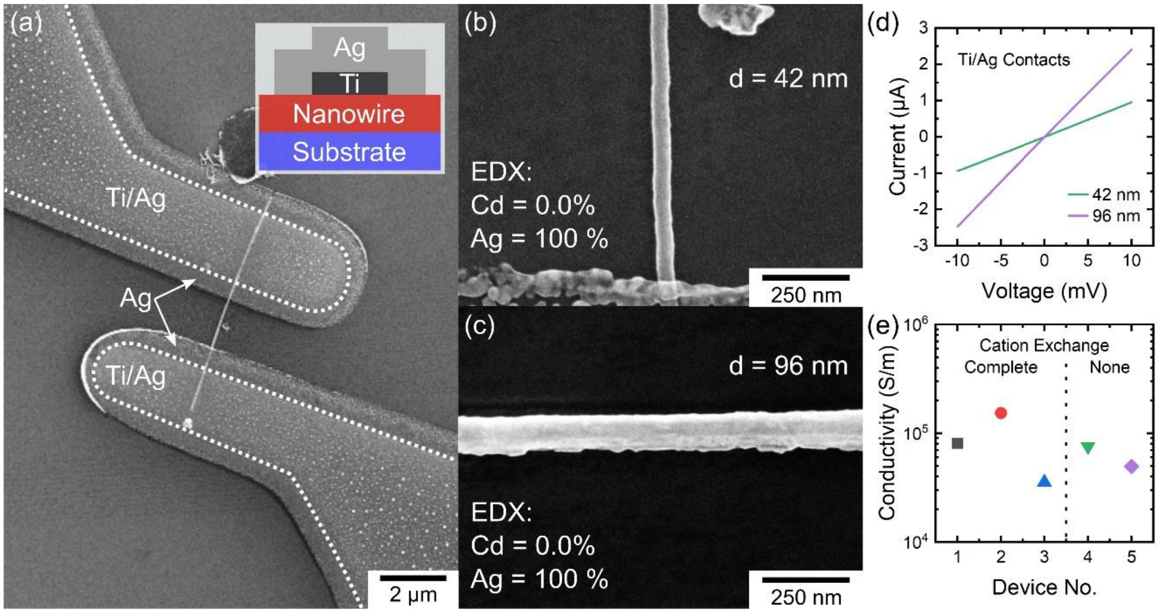

Figure S6. (a) SEM image of a device fabricated with Ti/Ag (10/50) nm contacts. The dashed line indicates the regions where both metals are present and where only silver is left. The inset schematically shows the metal contact profile (b-c) Higher magnification SEM images of the NWs. EDX was performed on this fraction of the NW and the determined cadmium and silver content is noted. Additionally, the diameter measured by AFM is given. (d) Current-voltage curves of the two devices shown. (e) The obtained conductivity of these devices (No. 4 and 5) is compared to the devices in the manuscript (No. 1–3) after complete cation exchange with Ti 75 nm contacts.

To determine the influence of annealing, the devices were measured directly after metal deposition. In these measurements the devices showed a very high conductivity, as shown in figure S6 d. In fact, the obtained conductivity was already in the range of the fully exchanged NWs (shown in the manuscript), the comparison can be seen in figure S6 e. Elemental analysis of the NWs with EDX revealed that they had already full converted to Ag_2Se, as only Ag and no Cd could be detected. The measured fraction of the devices is shown in figure S6 b and c, with the corresponding determined elemental content. Most likely heating due to the deposition process, especially during Ti deposition, enabled the diffusion of silver. This again proves our hypothesis

that silver ions are very mobile in the CdSe lattice and also demonstrates the challenges of contact engineering.

References

(1) Grebinski, J. W.; Hull, K. L.; Zhang, J.; Kosel, T. H.; Kuno, M. Solution-Based Straight and Branched CdSe Nanowires. *Chem. Mater.* **2004**, *16*, 5260–5272.

(2) Dorn, A.; Wong, C. R.; Bawendi, M. G. Electrically Controlled Catalytic Nanowire Growth from Solution. *Adv. Mater.* **2009**, *21*, 3479–3482.

(3) Harder, P.; Nielsen, A.; Sassnau, A.-K.; Bonatz, D.; Perbandt, M.; Kipp, T.; Mews, A. Determination of the Wurtzite and Zincblende Fractions in II–VI Semiconductor Nanowires. *Chem. Mater.* **2021**, *33*, 1061–1069.

(4) Sowa, H. The High-Pressure Behaviour of CdSe up to 3 GPa and the Orientation Relations Between its Wurtzite- and NaCl-Type Modifications. *Solid State Sciences* **2005**, *7*, 1384–1389.

(5) Yu, J.; Yun, H. Reinvestigation of the Low-Temperature form of Ag_2Se (Naumannite) Based on Single-Crystal Data. *Acta Crystallographica Section E* **2011**, *67*, i45.

(6) Li, H.; Zanella, M.; Genovese, A.; Povia, M.; Falqui, A.; Giannini, C.; Manna, L. Sequential Cation Exchange in Nanocrystals: Preservation of Crystal Phase and Formation of Metastable Phases. *Nano Lett.* **2011**, *11*, 4964–4970.

(7) Dorn, A.; Allen, P. M.; Harris, D. K.; Bawendi, M. G. In Situ Electrical Monitoring of Cation Exchange in Nanowires. *Nano Lett.* **2010**, *10*, 3948–3951.

(8) Schwarz, M.; Mews, A.; Dorn, A. Superionic Phase Transition in Individual Silver Selenide Nanowires. *Nanoscale* **2021**, *13*, 8017–8023.

(9) Kuno, M. An Overview of Solution-Based Semiconductor Nanowires: Synthesis and Optical Studies. *Phys. Chem. Chem. Phys.* **2008**, *10*, 620–639.

(10) Miszta, K.; Dorfs, D.; Genovese, A.; Kim, M. R.; Manna, L. Cation Exchange Reactions in Colloidal Branched Nanocrystals. *ACS Nano* **2011**, *5*, 7176–7183.

(11) Butterfield, A. G.; Alameda, L. T.; Schaak, R. E. Emergence and Control of Stacking Fault Formation during Nanoparticle Cation Exchange Reactions. *J. Am. Chem. Soc.* **2021**, *143*, 1779–1783.

(12) Gates, B.; Wu, Y.; Yin, Y.; Yang, P.; Xia, Y. Single-Crystalline Nanowires of Ag_2Se Can Be Synthesized by Templating against Nanowires of Trigonal Se. *J. Am. Chem. Soc.* **2001**, *123*, 11500–11501.

(13) Khanal, D. R.; Wu, J. Gate Coupling and Charge Distribution in Nanowire Field Effect Transistors. *Nano Lett.* **2007**, *7*, 2778–2783.

(14) Wunnicke, O. Gate Capacitance of Back-Gated Nanowire Field-Effect Transistors. *Appl. Phys. Lett.* **2006**, *89*, 83102.

(15) Deslattes, R. D.; Kessler, E. G.; Indelicato, P.; Billy, L. de; Lindroth, E.; Anton, J. X-ray Transition Energies: New Approach to a Comprehensive Evaluation. *Rev. Mod. Phys.* **2003**, *75*, 35–99.

(16) Ng, K. K.; Sze, S. M., Eds. *Physics of Semiconductor Devices 3rd Edition*; John Wiley & Sons Incorporated, 2006.

(17) Nouchi, R. Extraction of the Schottky Parameters in Metal-Semiconductor-Metal Diodes from a Single Current-Voltage Measurement. *J. Appl. Phys.* **2014**, *116*.

(18) Hajzus, J. R.; Biacchi, A. J.; Le, S. T.; Richter, C. A.; Hight Walker, A. R.; Porter, L. M. Contacts to Solution-Synthesized SnS Nanoribbons: Dependence of Barrier Height on Metal Work Function. *Nanoscale* **2017**, *10*, 319–327.

(19) Zhang, Z.; Yao, K.; Liu, Y.; Jin, C.; Liang, X.; Chen, Q.; Peng, L.-M. Quantitative Analysis of Current–Voltage Characteristics of Semiconducting Nanowires: Decoupling of Contact Effects. *Adv. Funct. Mater.* **2007**, *17*, 2478–2489.

(20) Junod, P. Relations Entre la Structure Cristalline et Les Propriétés Électroniques des Combinaisons Ag-2S, Ag-2Se, Cu-2Se, ETH Zurich, 1959.

(21) Wen, J.; Zhang, X.; Gao, H.; Wang, M. Current–Voltage Characteristics of the Semiconductor Nanowires Under the Metal-Semiconductor-Metal Structure. *J. Appl. Phys.* **2013**, *114*, 223713.
